# New Anti-Cancer Impact of Cerium Oxide, Lithium, and Sn-38 Synergy via DNA Methylation-Mediated Reduction of MMP-2 and Modulation of the PI3K/Akt/mTOR Pathway

**DOI:** 10.3390/ph18111725

**Published:** 2025-11-13

**Authors:** Sidika Genc, Hayrunnisa Nadaroglu, Ramazan Cinar, Esmanur Nigde, Kubra Karabulut, Ali Taghizadehghalehjoughi

**Affiliations:** 1 Department of Medical Pharmacology, Faculty of Medicine, Bilecik Şeyh Edebali University, 11230 Bilecik, Turkey; esmanurnigde123@gmail.com (E.N.); kubrakarabulut75@gmail.com (K.K.); 2 Department of Food Technology, Vocational College of Technical Science, Ataturk University, 25240 Erzurum, Turkey; hnisa25@atauni.edu.tr; 3 Department of Nanoscience and Nano-Engineering, Institute of Science and Technology, Ataturk University, 25240 Erzurum, Turkey; 4 Department of Biophysics, Faculty of Medicine, Bilecik Şeyh Edebali University, 11230 Bilecik, Turkey; ramazan.cinar@bilecik.edu.tr

**Keywords:** anti-cancer, cerium oxide, glioblastoma, lithium, SN-38

## Abstract

**Background/Objectives:** Glioblastoma, the most common primary tumor of the central nervous system, is characterized by high malignancy and poor prognosis. One of the main challenges in neurological disorders is to develop an effective treatment modality that can cross the blood–brain barrier. Nanoparticles are revolutionary for neurodegenerative diseases due to their targeted delivery and ability to overcome biological barriers. Cerium oxide (Ce_2_O_3_) nanoparticles are suitable for use as drug delivery systems. **Methods:** In our study, we investigated the anticancer mechanism using SN-38, lithium, and Ce_2_O_3_, a powerful agent used in GBM treatment. We evaluated their anticancer activities separately and in combination with U373 cell lines. GBM cell line U373 cells were cultured. Then, all groups except the control group were treated with different doses of SN-38 and lithium combination therapy with SN-38, lithium, and Ce_2_O_3_ combination therapy. The results were evaluated using MTT and ELISA tests. **Results:** When the results were examined, anticancer activity was detected at PTEN, AKT, mTOR, and BAX/Bcl-2 levels in the SN-38 + NPs 25 µg/mL + Lithium 50 µg/mL and SN-38 + NPs 50 µg/mL + Lithium 50 µg/mL dose groups. In addition, findings that inflammation markers were correlated with the apoptosis mechanism were obtained. **Conclusion:** This study is the first to report that combining lithium with SN-38 and NPs increased oxidative stress more than lithium with SN-38, leading glioblastoma cells to apoptosis and its potential anticancer activity. These results provide a basis for further investigation of its clinical application in cancer treatment.

## 1. Introduction

Cancer is a disease characterized by the uncontrolled proliferation of specific cells. The hallmarks of cancer include sustained proliferative signaling, evasion of growth suppressors, activation of metastasis, induction of mutations, resistance to cell death, and dysregulation of cellular metabolism [1]. Glioblastoma (GBM) is the most common primary malignant brain tumor, accounting for approximately 16% of all central nervous system neoplasms. The incidence rate is about 3.2 cases per 100,000 individuals, with a median survival time of 15 months [2,3]. GBM is particularly difficult to treat due to its highly heterogeneous structure, necessitating the use of multiple therapeutic strategies in combination [4,5]. The multimodal treatment approaches for GBM include surgery, radiotherapy, chemotherapy, and combined therapies [3,4,6].

SN-38 (7-ethyl-10-hydroxycamptothecin), a potent anticancer agent derived from camptothecin (CPT-11), is widely used in the treatment of GBM and acts as a topoisomerase I inhibitor [7,8]. SN-38 interacts with the DNA–topoisomerase I complex to form a stable ternary complex that prevents DNA re-ligation, disrupts the replication fork, induces replication arrest, causes DNA strand breaks, and ultimately leads to cell death [8]. In addition to anticancer agents, various adjuvant compounds and metabolites are also employed in GBM therapy. In our study, a combined treatment of lithium and SN-38 was applied, and their anticancer activities were investigated in U373 glioblastoma cells.

Lithium (Li) is a monovalent cation from the alkali metals group, with an atomic number of 3 [9]. Li generates the bioactive form of lithium via its interaction with ATP and Mg^2+^, which subsequently influences purinergic receptor activation in neurons. Li specifically inhibits the coupling of β-adrenergic and muscarinic receptors to G proteins, a process facilitated by magnesium. It also restricts the activity of inositol monophosphatase, an enzyme that requires magnesium, and inhibits bisphosphate 3-prime-nucleotidase (BPNT1), a magnesium-dependent phosphatase [10]. Li has been used for decades in the treatment of bipolar disorder, prevention of relapses, and prophylaxis of manic episodes. The neuroprotective effect of lithium is linked to the suppression of its molecular targets, particularly inositol monophosphatase (IMPase), glycogen synthase kinase 3β (GSK3), and protein kinase C (PKC) [11,12]. Interestingly, these enzymes are dysregulated in cancer, suggesting a potential role for Li in cancer treatment beyond bipolar disorder [13,14]. Recent in vitro and in vivo studies have reported the effects of lithium on cell proliferation, tumor growth, and cancer metabolism [13,15]. According to this evidence, Li inhibited cell proliferation in colon, breast, pancreatic, and neuroblastoma cancers [16]. Additionally, the chlorinated form of lithium inhibits glycogen synthase kinase 3β (GSK3β), a serine/threonine protein kinase [10,12]. GSK3β phosphorylates β-catenin within the Wnt signaling pathway, leading to growth arrest. Additionally, GSK3β is involved in cellular proliferation and survival by activating nuclear factor κB-dependent gene transcription, suggesting its significant role in tumorigenesis in pancreatic, colorectal, and prostate cancers, as well as gliomas [16,17].

Lithium’s anti-inflammatory efficacy stems from its strong inhibitory effect on GSK3β, reduced activation of the NF-κB and JNK signaling pathways, and decreased TNF-α production through stimulation of the cytokine IL-10 [12,18]. The anti-inflammatory activity of lithium is attributed to the following mechanisms: diminished cyclooxygenase-2 expression, increased nitric oxide synthase expression, suppression of interleukin-1β and TNF-α, and enhanced synthesis of IL-2 and IL-10. Given all this evidence, lithium is believed to have a protective effect on organs during tumor development. [15,19].

A primary challenge in neurological diseases is developing effective medications that can cross the blood–brain barrier [20]. Nanoparticles are revolutionary for treating neurodegenerative conditions because of their precise delivery and ability to bypass biological barriers. Cerium oxide nanoparticles (Ce_2_O_3_ NPs) have garnered significant attention due to their biological applications, particularly for their antioxidant properties. Ce_2_O_3_ nanoparticles can reversibly bind oxygen and switch between oxidation states, allowing them to eliminate excess reactive oxygen species (ROS) in tissues [21]. They also exhibit antioxidant activity, including the ability to mimic the functions of superoxide dismutase (SOD) and catalase enzymes. Unlike traditional antioxidants, the radical scavenging ability of Ce_2_O_3_ nanoparticles is regenerative, enabling longer-lasting activity [22].

Recent evidence indicates that targeting multiple oncogenic and redox-sensitive signaling pathways simultaneously can overcome chemoresistance and improve outcomes in solid tumors. Based on this, we developed a new combination approach that includes SN-38, lithium, and cerium oxide nanoparticles (Ce_2_O_3_ NPs), leveraging their complementary molecular actions. SN-38, a topoisomerase I inhibitor and the active metabolite of irinotecan, causes DNA damage and triggers apoptosis, though its effectiveness is often limited by systemic toxicity and resistance. Lithium inhibits tumor growth by inhibiting GSK-3β, thereby downregulating the PI3K/Akt/mTOR pathway [23]. Ce_2_O_3_ NPs, through their unique Ce^3+^/Ce^4+^ redox cycling, modulate ROS production and oxidative stress within the tumor microenvironment [24,25]. These agents were selected to act synergistically: lithium suppresses survival signaling, thereby increasing SN-38-induced apoptosis; Ce_2_O_3_ NPs further sensitize tumor cells through oxidative regulation, which impacts DNA methylation. This strategy aims to reduce MMP-2 expression and inhibit the PI3K/Akt/mTOR pathway through mechanisms related to DNA methylation.

GBM remains one of the most aggressive and treatment-resistant brain tumors, where conventional chemotherapy often fails due to toxicity and adaptive resistance. While SN-38, lithium, and Ce_2_O_3_ have each shown promising biological effects individually—as a DNA topoisomerase inhibitor, GSK-3β suppressor, and redox modulator, respectively—their combined molecular synergy has not been investigated. No previous studies have evaluated the joint impact of SN-38, lithium, and Ce_2_O_3_ NPs on DNA methylation, oxidative stress, and the PI3K/Akt/mTOR signaling cascade in GBM.

Therefore, this study aims to fill this gap by developing a novel, mechanism-based combinatorial approach using SN-38, lithium, and Ce_2_O_3_ NPs to enhance therapeutic efficacy while minimizing cytotoxicity. The present work proposes, for the first time, a synergistic model in which Ce_2_O_3_ NPs stabilize redox homeostasis, lithium suppresses oncogenic PI3K/Akt/mTOR signaling, and SN-38 induces apoptosis via DNA-methylation-mediated modulation of MMP-2 and pro-apoptotic genes. Accordingly, our study investigated the anticancer effects of combining SN-38, lithium, and Ce_2_O_3_ nanoparticles on the U373 GBM cell line. After 24 h of incubation with different doses of SN-38-Lit and SN-38-Lit-NPs, cell viability and oxidative stress parameters (MTT, TAC, TOS) were examined. In addition, apoptotic markers (*Caspase-3*, *Caspase-9*, *BAX*, *BCL-2*, *p21*, *p53*, PTEN, AKT, mTOR, STAT-3, and MMP-2) were analyzed to confirm cellular apoptosis, and inflammation markers were evaluated to determine potential anti-inflammatory effects.

## 2. Results

### 2.1. Cerium Oxide Characterization

**Figure 1:**
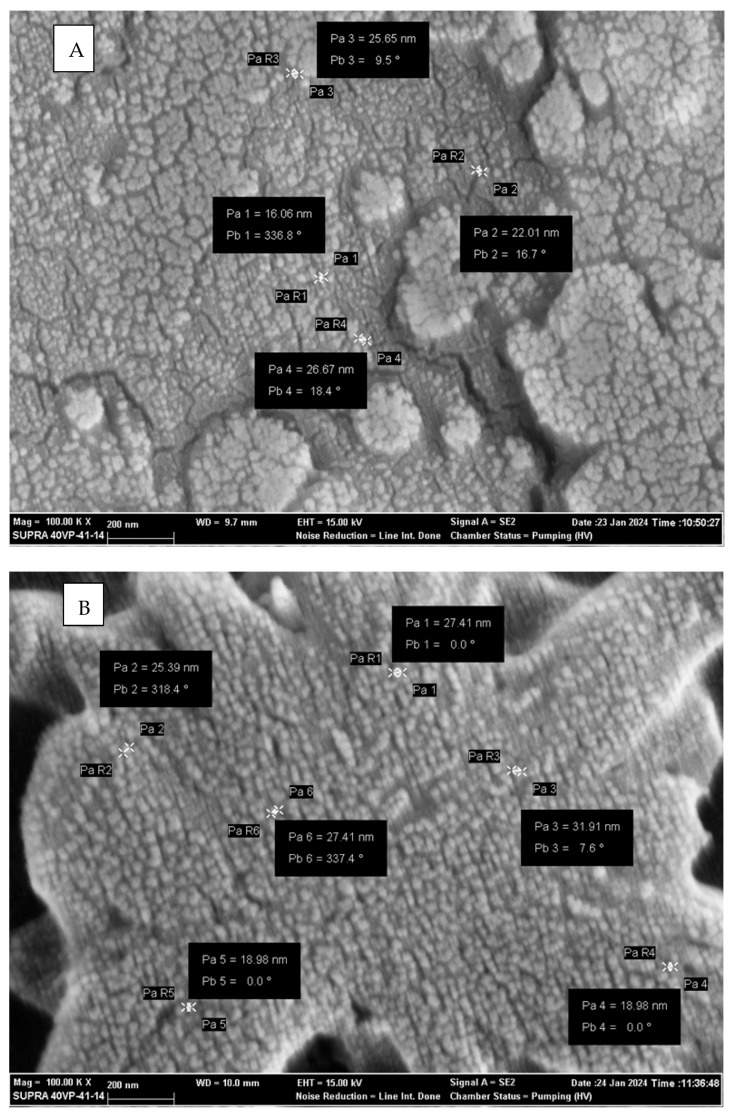
(**A**). Ce_2_O_3_ SEM results; (**B**). Lithium and Sn38-loaded Ce_2_O_3_ NPs SEM results. Analysis of exosome sizes by SEM. The white arrow indicates particle size. Pa: particle size (nm), P: particle, Pa: particle radius, and Pb: particle angle (Carl Zeiss Evo 40 SEM (Jena, Germany) was used to acquire the images).

Scanning Electron Microscopy (SEM) analysis was performed to determine the morphological properties and surface topology of Ce_2_O_3_ nanoparticles (NPs) synthesized via the hydrothermal method. The surface morphology was examined using a ZEISS Supra 40 VP Scanning Electron Microscope (Oberkochen, Germany) at 100,000× magnification, and representative micrographs are presented in Figure 1A.

As seen in the images, the Ce_2_O_3_ NPs exhibit a spherical to subspherical morphology with a uniform distribution and minimal aggregation, indicating the successful dispersion and stability of the synthesized nanoparticles. The particle size was estimated to be between 16.06 nm and 58.17 nm, consistent with the nanoscale size required for biomedical applications. The relatively narrow size distribution suggests that the hydrothermal synthesis route effectively controls the nucleation and growth of the nanoparticles, resulting in homogeneous morphology. Particle size distribution was further confirmed by Dynamic Light Scattering (DLS) analysis (Figure 1B).

**Figure 2:**
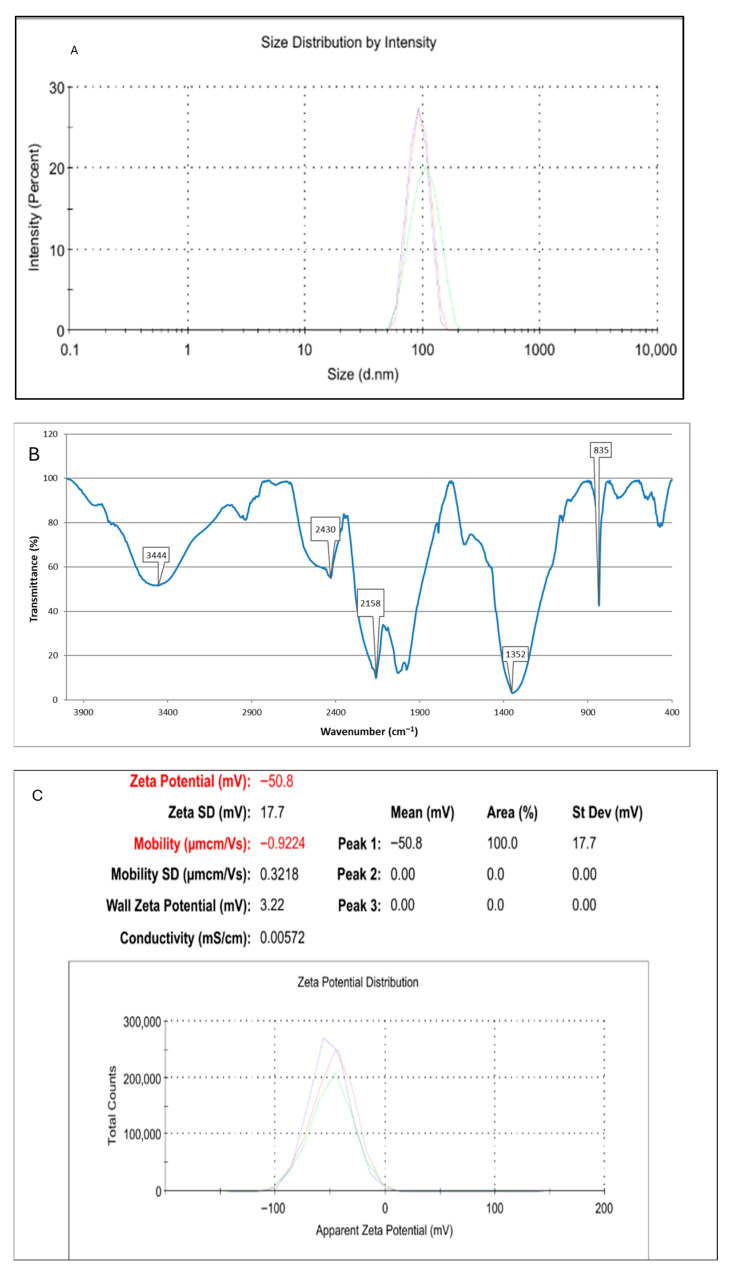

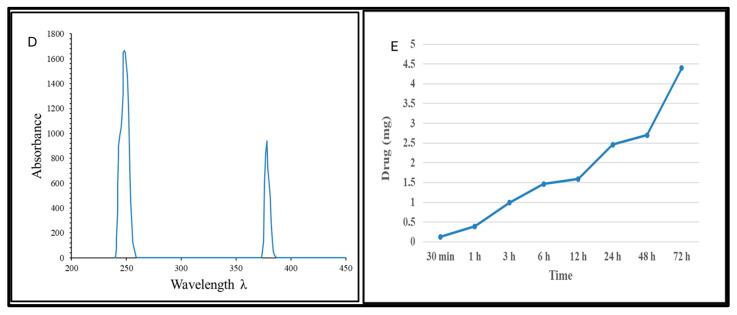
(**A**) DLS and (**B**) FT-IR and (**C**) Zeta potential results of Ce_2_O_3_ NPs. (**D**) Absorbance data of the synthesized bioconjugated drug were determined by a UV-Vis spectrophotometer. (**E**) Cumulative release profile graph of bioconjugated NPs.

DLS analysis showed that the synthesized Ce_2_O_3_ nanoparticles had a Z-average diameter of 213.4 nm and a polydispersity index (PDI) of 0.380, indicating a moderately homogeneous size distribution. The dominant peak was observed at 83.83 nm, representing 100% of the total particle population, and had a standard deviation of 19.34 nm. The high intercept value (0.956) confirms the good correlation quality and reliability of the measurement. From a biomedical perspective, nanoparticles with diameters below 100 nm are generally considered capable of crossing the blood–brain barrier (BBB) through mechanisms such as adsorption-mediated or receptor-mediated transcytosis. In this study, although the Z-average value indicates some aggregation, the main population, centered around 84 nm, falls within the BBB-permeability range. This suggests that these Ce_2_O_3_ nanoparticles could potentially cross the blood–brain barrier and be used in neurotherapeutic or brain-targeted drug delivery applications. Overall, the results confirm that the synthesized Ce_2_O_3_ nanoparticles have nanometer-scale dimensions, moderate monodispersity, and a particle fraction compatible with BBB permeability, making them promising candidates for therapeutic studies related to neurological and oxidative stress (Figure 2A).

The FTIR spectrum of the synthesized Ce_2_O_3_ nanoparticles reveals characteristic bands specific to the metal oxide structure, as well as the presence of some functional groups adsorbed on the surface. The broad band around 3400 cm^−1^ corresponds to O–H stretching vibrations and indicates the presence of hydroxyl groups or water molecules adsorbed to the surface. The weak band around 1630 cm^−1^ is associated with H–O–H bending vibrations. Peaks in the 1450–1400 cm^−1^ range represent carbonate (CO_3_^2−^) stretching vibrations, suggesting partial carbonation of the surface by atmospheric CO_2_. Bands around 1050 cm^−1^ may indicate Ce–O–C or Ce–O–H bonds originating from organic stabilizers such as citrate used during synthesis. The most prominent region of the spectrum, the band between 500 and 600 cm^−1^, corresponds to Ce–O lattice vibrations and confirms the formation of the Ce_2_O_3_ crystalline phase. The broad peak profile in this region indicates that the material has a nanocrystalline structure. FTIR analysis confirms that the synthesized Ce_2_O_3_ nanoparticles have a metal oxide structure containing hydroxyl and carbonate groups on the surface and that they form in harmony with the characteristic Ce–O vibrations (Figure 2B).

The zeta potential of the resulting Ce_2_O_3_ nanoparticles has been evaluated using a MALVERN Nano-ZS Zetasizer (Malvern, UK), with the findings illustrated in Figure 2C The nanoparticles demonstrated an average zeta potential of −50.8 mV, with an average deviation of ±17.7 mV and a distinct peak at this value, signifying a consistent surface charge distribution. The elevated negative zeta potential indicates that the Ce_2_O_3_ nanoparticles exhibit substantial electrostatic repulsion among particles, inhibiting aggregation and ensuring superior colloidal stability in aqueous suspension. Zeta potential values surpassing ±30 mV are indicative of a highly stable nanoparticle system; thus, the detected −50.8 mV corroborates the efficacy of the synthesis and washing techniques in yielding stable and well-dispersed nanoparticles.

The graph obtained from spectrophotometric measurements related to drug packaging into nanoparticles is given in Figure 2. As a result of absorbance measurements performed with a UV-visible spectrophotometer, which were carried out separately for each application, the absorbance wavelengths of the bioconjugate material (Sn 10 nM + NPs 50 µg/mL + Lit 50 µg/mL) were determined to be 240 and 260 nm. According to the spectrophotometric measurement performed at 248 nm to determine the amount of free drug after the bioconjugation process, no free drug was detected in the reaction solution (Figure 2D).

The cumulative graph obtained from spectrophotometric measurements performed to determine the release profile of the resulting bio-conjugated drug is shown in Figure 2E. According to this graph, 1.59 mg of drug was released from the bio-conjugated drug in the first 12 h, 2.46 mg at 24 h, and 2.77 mg at 48 h. The amount of drug released at the end of 72 h was recorded as 4.398 mg (87%).

### 2.2. Wound Assay Results

**Figure 3:**
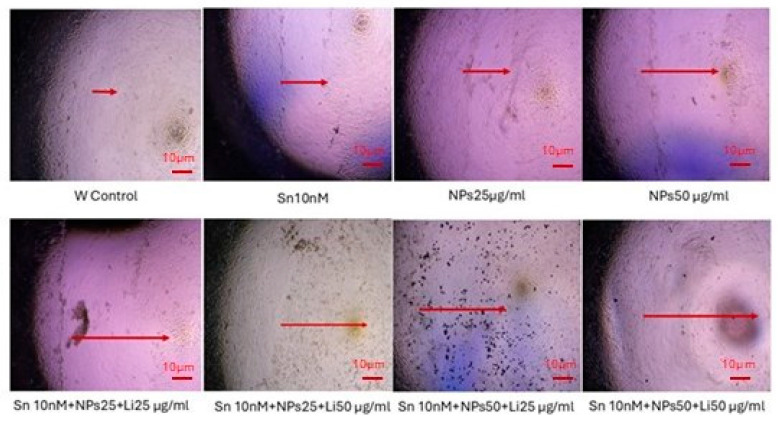
The wound test results evaluated the migration rate of the treatment groups in the U373 GBM cell line. Red arrow indicates the wound area.

In the migration test, in the cancerous U373 cell line, the wound opening did not close with treatment compared to the control, and apoptosis occurred. The most effective apoptosis occurred in the Sn 10 nM + NPs 50 µg/mL + Lit 25 µg/mL and Sn 10 nM + NPs 50 µg/mL + Lit 50 µg/mL dose groups compared to the control (Figure 3).

### 2.3. MTT Analysis Results

**Figure 4:**
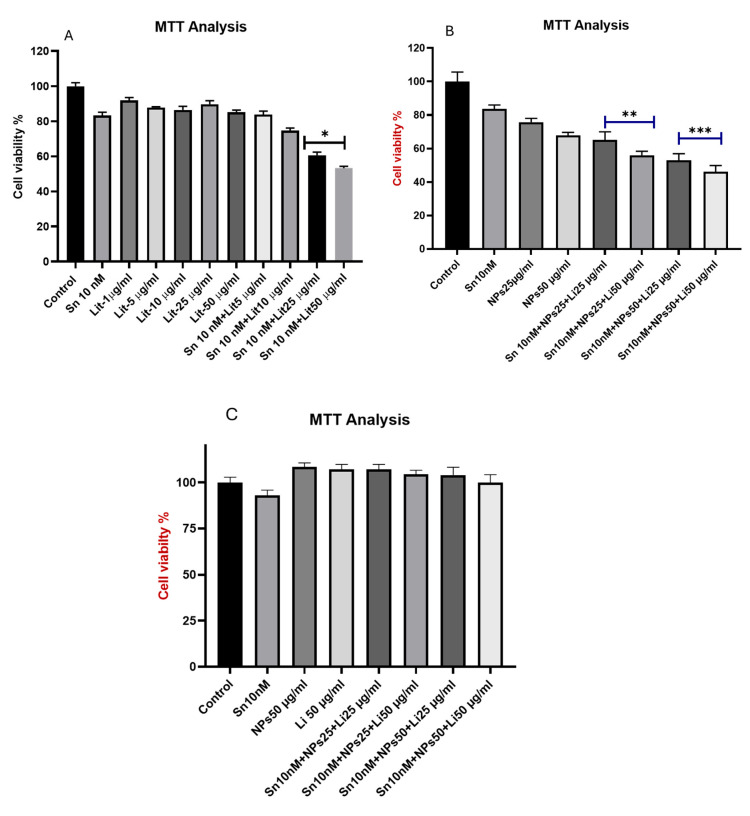

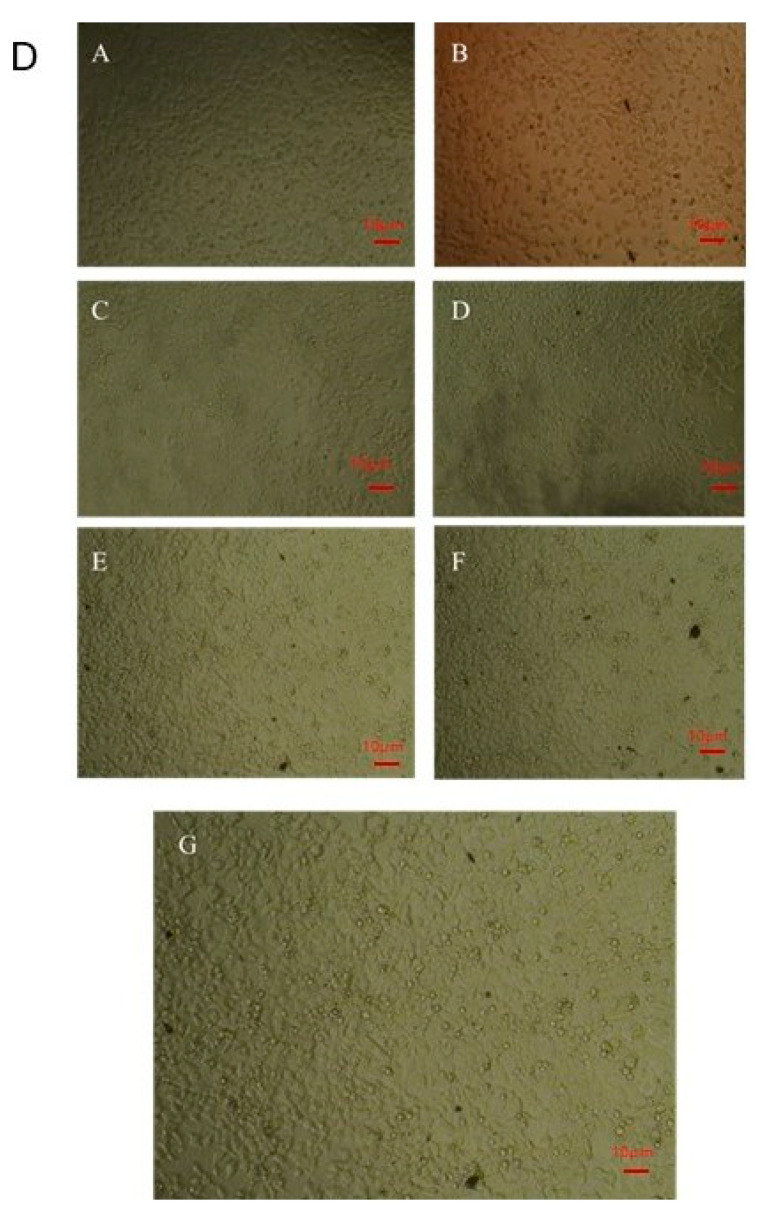
Cell viability of U373 cells at 24 h. (**A**). A significant difference was found in Sn 10 nM + Lit 25 µg/mL, and Sn 10 nM + Lit 50 µg/mL dose groups compared to the control. (**B**). The significance level was determined by comparing the dose groups Sn 10 nM + NPs 25 µg/mL + Lit 25 µg/mL and Sn 10 nM + NPs 25 µg/mL + Lit 50 µg/mL with the control. The significance level was determined as Sn 10 nM + NPs 50 µg/mL + Lit 25 µg/mL, Sn 10 nM + NPs 50 µg/mL + Lit 50 µg/mL compared to the control group (* *p* < 0.05, ** *p* < 0.01, *** *p* < 0.001). (**C**). Cell viability results of Neuroblastoma. (**D**). Microscope images of SHSY-5Y cells (A) Control; (B) Sn 10 nM, (C) Li 50, (D) NPs 50, (E) NPs 25 + Li 50 and Sn 10 nM, (F) NPs 50 + Li 25 and Sn 10 nM, (G) NPs 50 + Li 50 and Sn 10 nM.

The anticancer effects of SN-38 (10 nM), Lithium (1, 5, 10, 25, 50 µg/mL), and Cerium Oxide (25, 50 µg/mL) on U373 GBM cells were assessed after 24 h using the MTT assay. In Figure 1A,B, the spectrophotometric measurements of the control group were set to 100%, and the other groups were compared to the control. Results showed a 17% decrease in cell viability in the groups treated with a single dose of 10 nM Sn. A significant reduction was observed in the dose groups receiving Cerium Oxide compared to SN-38. In the lithium and NPs groups, cell viability decreased with increasing doses, but these changes were not statistically significant. In the combination treatments, the most notable results appeared in the groups of Sn 10 nM + Lit 25 µg/mL, Sn 10 nM + NPs 50 µg/mL + Lit 50 µg/mL, and Sn 10 nM + Lit 50 µg/mL. These findings indicate that cell viability was higher in the Sn 10 nM + NPs 50 µg/mL + Lit 50 µg/mL group than in the Sn 10 nM + Lit 50 µg/mL group (Figure 4A,B). Additionally, we examined the neurotoxic effects of this combination on SH-SY5Y neuroblastoma cells, which display neuron-like properties. Our results showed no cytotoxic effects on cell viability in either treatment group. Interestingly, cell proliferation was significantly increased in these groups (Figure 4C,D).

### 2.4. TAC and TOS Test Results

**Figure 5:**
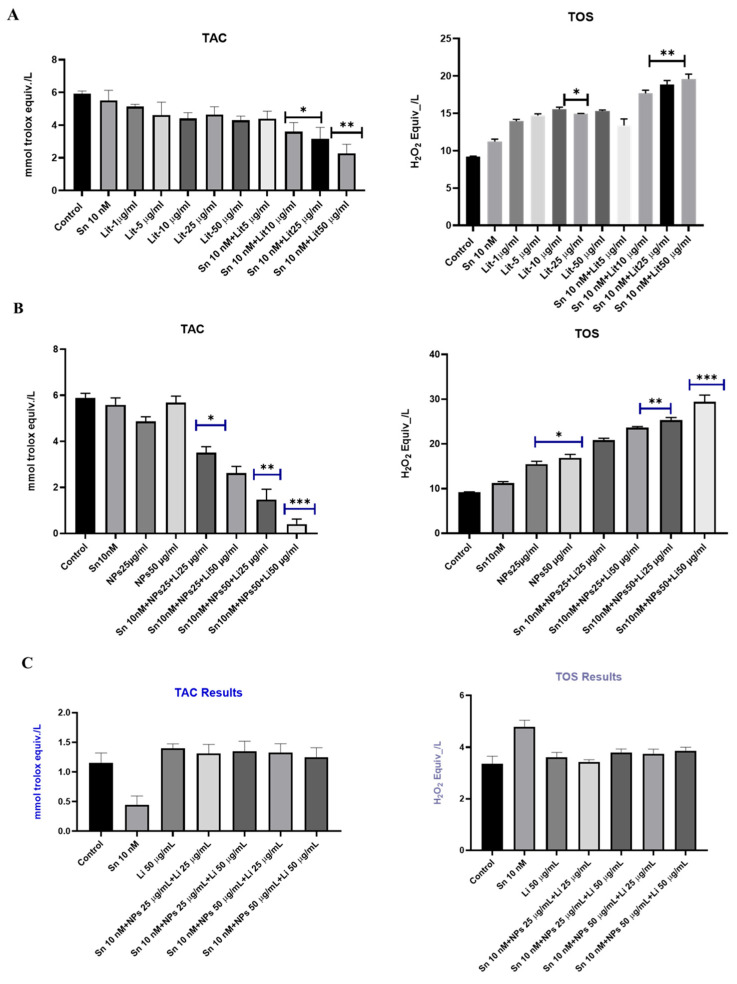
TAC and TOS levels in the control, Sn-38, NP, and treatment groups. (**A**) Sn-Li TAC and TOS results in U373; (**B**) Sn-NPs-Li TAC and TOS results in U373; (**C**) Sn-NPs-Li Sn-NPs-Li TAC and TOS results in SHSY-5Y. The Sn 10 nM + NPs 25 µg/mL + Lit 25 µg/mL group shows a significant difference compared to the control. The Sn 10 nM + NPs 50 µg/mL + Lit 25 µg/mL and Sn 10 nM + NPs 50 µg/mL + Lit 50 µg/mL groups are the most significantly different from the control. NPs in the 25 and 50 µg/mL dose groups show significant differences compared to the control. In combined applications, Sn 10 nM + NPs 25 µg/mL + Lit 50 µg/mL and Sn 10 nM + NPs 50 µg/mL + Lit 25 µg/mL groups also show significance compared to the control. The greatest increase is observed in the Sn 10 nM + NPs 50 µg/mL + Lit 50 µg/mL group compared to the control. No significant difference was observed in SHSY-5Y cells compared to the control (* *p* < 0.05, ** *p* < 0.01, *** *p* < 0.001).

To evaluate oxidative damage, TAC and TOS levels were measured from cell media collected after 24 h; all results are shown in Figure 5. A decrease in TAC levels was associated with reduced cell viability. The Sn, Li, and NPs groups, when used alone, showed minimal effects on oxidative damage. While Sn 10 nM + Lit 50 µg/mL demonstrated a significant reduction in TAC levels compared to the control group (about 61.82%), the most notable results were in the group with Sn 10 nM + NPs 50 µg/mL + Lit 50 µg/mL (around 40.44%). These findings suggest that antioxidant activity decreased as cell viability declined in U373 cells. No significant difference was observed in SHSY-5Y cells compared to the control. Conversely, oxidative stress increased at the cellular level, with a significant rise in TOS levels. Notably, the highest increase (approximately 219.84%) was seen in the group with Sn 10 nM + NPs 50 µg/mL + Lit 50 µg/mL. No significant difference was observed in SHSY-5Y cells compared to the control. Our results indicate that combining Sn 38 with lithium and cerium oxide made cancer cells more hypoxic, promoting apoptosis, compared to combining Sn 38 with lithium alone. Additionally, it has been observed that this combination does not induce oxidative stress in healthy neuronal-like cells.

### 2.5. ELISA Test Results

At the end of the experiment, BAX, BCL-2, PTEN, AKT, mTOR, STAT-3, MMP-2, IL-10, and IL-1β were assessed to determine levels of apoptosis, cell proliferation, and inflammation. The most used parameters in cellular apoptotic mechanisms include measuring BAX/Bcl-2 levels and PTEN, as well as AKT, mTOR, and STAT-3 levels. Therefore, we measured BAX, Bcl-2, PTEN, AKT, mTOR, and STAT-3 protein levels spectrophotometrically to evaluate the effects of Sn + Lit and Sn + NPs + Lit combinations.

**Figure 6:**
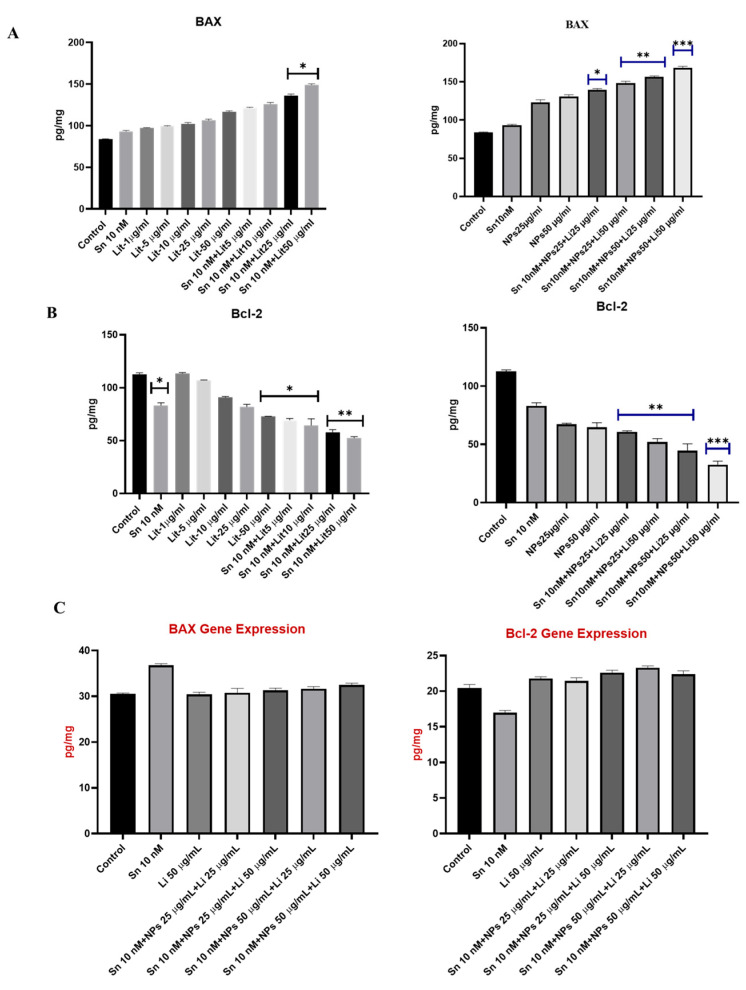
BAX and BCL-2 levels of control, Sn-38, NPs, and treatment groups. (**A**) BAX result; Sn 10 nM + Lit 25 µg/mL, Sn 10 nM + Lit 50 µg/mL, and Sn 10 nM + NPs 25 µg/mL + Lit 25 µg/mL, Sn 10 nM + NPs 25 µg/mL + Lit 50 µg/mL, and Sn 10 nM + NPs 50 µg/mL + Lit 25 µg/mL treatment groups are significant compared to the control. Compared to the control, the highest significance is observed in the Sn 10 nM + NPs 50 µg/mL + Lit 50 µg/mL dose group. (**B**) BCL-2 result; Among the combined treatment groups, Sn 10 nM, Lit 25 and 50 µg/mL, and Sn 10 nM + Lit 5 µg/mL dose groups are significant compared to the control. In the combined treatment groups, Sn 10 nM + Lit 10 µg/mL, Sn 10 nM + Lit 25 µg/mL, Sn 10 nM + Lit 50 µg/mL, Sn 10 nM + NPs 25 µg/mL + Lit 25 µg/mL, Sn 10 nM + NPs 25 µg/mL + Lit 50, and Sn 10 nM + NPs 50 µg/mL + Lit 25 µg/mL were significant compared to the control. The highest significance rate compared to the control was observed in the Sn 10 nM + NPs 50 µg/mL + Lit 50 µg/mL dose group. (**C**) Results for BAX and Bcl-2 in SHSY-5Y cells. In both analyses, the dose groups showed no significant difference compared to the control groups (* *p* < 0.05, ** *p* < 0.01, *** *p* < 0.001).

As shown in Figure 6, BAX levels increased by 60% in the Sn 10 nM + Lit 25 µg/mL, Sn 10 nM + Lit 50 µg/mL, and Sn 10 nM + NPs 25 µg/mL + Lit 25 µg/mL treatment groups compared to the control. In the combined treatment groups Sn 10 nM + NPs 25 µg/mL + Lit 50 µg/mL and Sn 10 nM + NPs 50 µg/mL + Lit 25 µg/mL, an 86.63% increase in BAX levels was observed, accompanied by a decrease in cell viability. The most effective results were seen in the Sn 10 nM + NPs 50 µg/mL + Lit 50 µg/mL dose group, with an increase of approximately 100.63% compared to the control. Additionally, BAX levels in healthy neuron-like cells did not differ significantly from those in power. Our results indicate that although Sn-38 alone is apoptotic in cancer cells, its use in combination with lithium and cerium oxide is more effective. However, it was found to have no apoptotic effect in SHSY-5Y cells. A significant decrease in the anti-apoptotic marker BCL-2 level was found in the Sn 10 nM, Lit 25 and 50 µg/mL, and Sn 10 nM + Lit 5 µg/mL dose groups compared to the control (* *p* < 0.05). Among the treatment groups, the most significant decreases were observed in the Sn 10 nM + Lit 50 µg/mL (53.57%) and Sn 10 nM + NPs 50 µg/mL + Lit 50 µg/mL (approximately 71%) dose groups. Additionally, Bcl-2 levels in healthy neuron-like cells did not differ significantly from those in the control (Figure 6).

**Figure 7:**
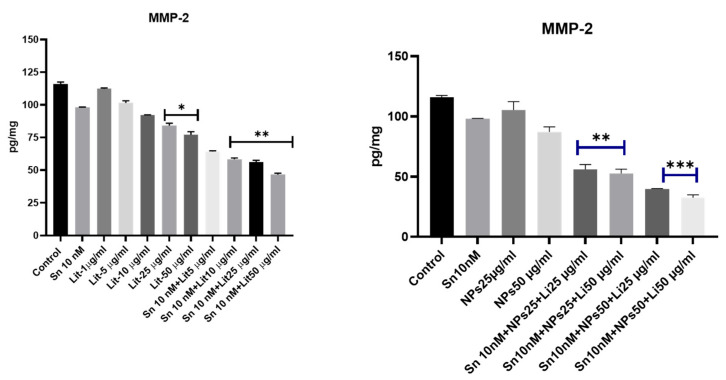
MMP-2 levels of control, Sn-38, NPs, and treatment groups. A significant decrease was observed in the Lit 25 µg/mL and Lit 50 µg/mL dose groups compared to the control. The highest reduction rate was detected in the combined treatment groups of Sn 10 nM + NPs 50 µg/mL + Lit 25 µg/mL, and Sn 10 nM + NPs 50 µg/mL + Lit 50 µg/mL compared to the control (* *p* < 0.05, ** *p* < 0.01, *** *p* < 0.001).

MMP-2 levels increase in proportion to cancer cell viability. Although Lithium combined with SN-38 in our study significantly reduced cell viability, the most effective results were observed when it was combined with serum oxide nanoparticles. Consequently, a significant reduction in MMP-2 protein levels was noted (Figure 7). Among the combined treatment groups, Sn 10 nM + Lit 10 µg/mL, Sn 10 nM + Lit 25 µg/mL, Sn 10 nM + Lit 50 µg/mL, Sn 10 nM + NPs 25 µg/mL + Lit 25 µg/mL, and Sn 10 nM + NPs 25 µg/mL + Lit 50 µg/mL showed significant differences compared to the control. The greatest decreases were observed in the combinations of Sn 10 nM + NPs 50 µg/mL + Lit 25 µg/mL and Sn 10 nM + NPs 50 µg/mL + Lit 50 µg/mL. This reduction in protein levels is linked to increased BAX levels and decreased cell viability.

**Figure 8:**
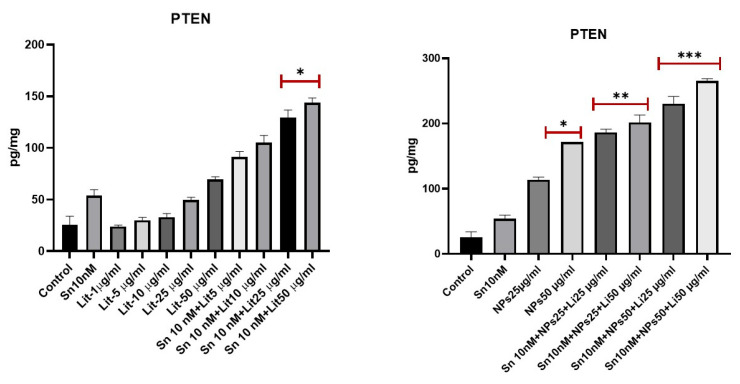
PTEN levels of control, Sn-38, NPs, and treatment groups. A significant increase was observed in Sn 10 nM + Lit 25 µg/mL, Sn 10 nM + Lit 50 µg/mL, and NPs 50 µg/mL dose groups compared to the control (* *p* < 0.05, ** *p* < 0.01, *** *p* < 0.001).

PTEN is a crucial activator of the PI3K signaling pathway within the cell. It plays an essential role in preventing cell growth and proliferation during the process of oncogenesis. An increase in PTEN levels was observed in the groups treated with SN-38 at 10 µg/mL + NPs at 50 µg/mL + Lit at 25 µg/mL and SN-38 at 10 µg/mL + NPs at 50 µg/mL + Lit at 50 µg/mL. This increase correlates with the levels of AKT and mTOR (Figure 8).

**Figure 9:**
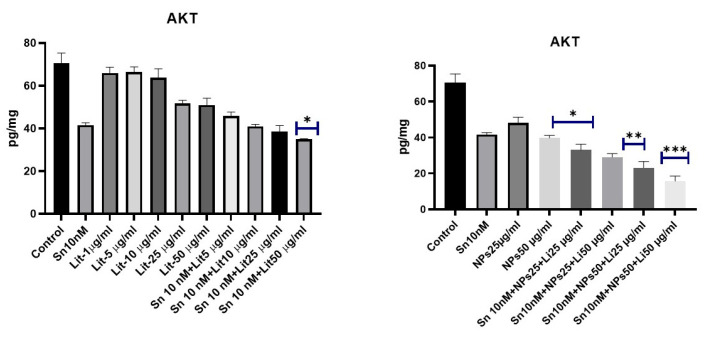
AKT levels across control, Sn-38, NPs, and treatment groups. A significant reduction was seen in the groups treated with Sn-38 10 nM + Lit 50 µg/mL, NPs 50 µg/mL, and Sn-38 10 nM + NPs 25 µg/mL + Lit 25 µg/mL compared to the control (* *p* < 0.05, ** *p* < 0.01, *** *p* < 0.001).

AKT, which plays a role in cell metabolism, growth, proliferation, and survival, acts as a crucial marker for anticancer activity. A significant decrease in AKT levels was seen in the groups treated with SN-38 10 nM + NPs 50 µg/mL + Lit 25 µg/mL and SN-38 10 nM + NPs 50 µg/mL + Lit 50 µg/mL. This result also aligns with other parameters (Figure 9).

**Figure 10:**
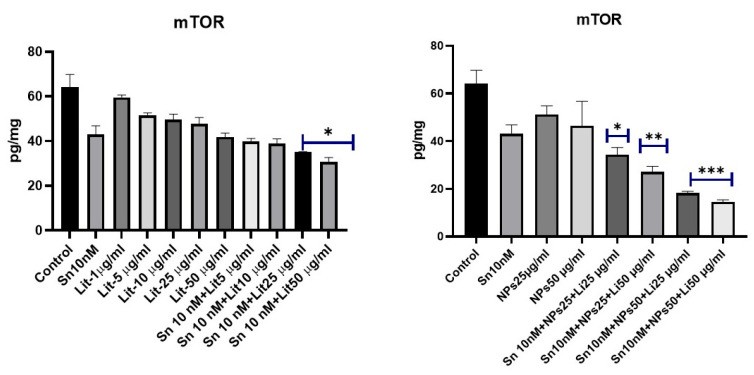
mTOR levels of control, Sn-38, NPs, and treatment groups. A decrease was observed in Sn 10 nM + Lit 50 µg/mL, and Sn 10 nM + NPs 25 µg/mL + Lit 25 µg/mL dose groups compared to the control (* *p* < 0.05, ** *p* < 0.01, *** *p* < 0.001).

**Figure 11:**
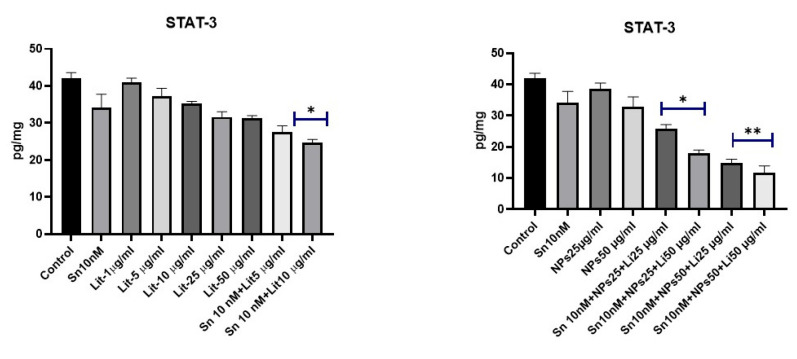
STAT-3 levels of control, Sn-38, NPs, and treatment groups. A decrease was observed in Sn 10 nM+Lit 50 µg/mL, Sn 10 nM+NPs 25 µg/mL + Lit 25 µg/mL, and Sn 10 nM+NPs 25 µg/mL + Lit 50 µg/mL dose groups compared to the control (* *p* < 0.05, ** *p* < 0.01).

The transcription factor STAT-3 also activates mTOR (Figure 10 and Figure 11). Both are involved in cellular metabolism and apoptosis. A significant decrease in STAT-3 levels was observed in the groups treated with SN-38 10 nM + NPs 50 µg/mL + Lit 25 µg/mL and SN-38 10 nM + NPs 50 µg/mL + Lit 50 µg/mL. This reduction is associated with other markers of apoptosis.

**Figure 12:**
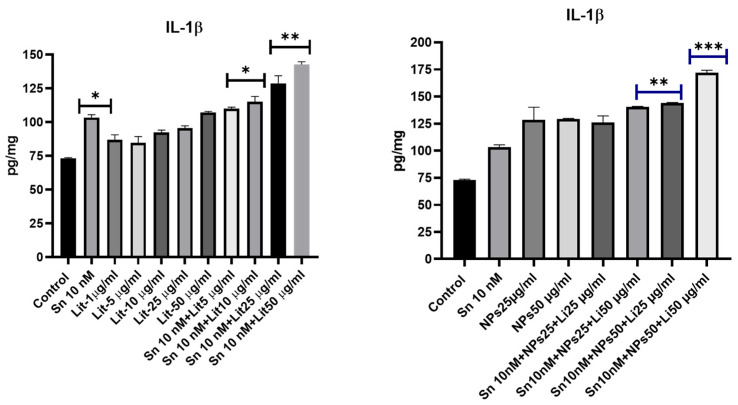
IL-1β levels in control, Sn-38, NPs, and treatment groups. Sn 10 nM, Sn 10 nM + Lit 5 µg/mL, and the dose group of Sn 10 nM + Lit 5 µg/mL show significant increases compared to the control. (* *p* < 0.05, ** *p* < 0.01, *** *p* < 0.001).

The level of inflammation in cells increased as cell viability decreased and the apoptosis mechanism was activated. Therefore, inflammation levels rose in the combined treatment groups, especially in the Sn 10 nM + Lit 25 µg/mL, Sn 10 nM + Lit 50 µg/mL, and Sn 10 nM + NPs 50 µg/mL + Lit 50 µg/mL groups (Figure 12).

### 2.6. Immunohistochemical Results

Annexin V-FITC analysis of U373 GBM cells treated with combinations of lithium, Sn-38, and cerium oxide

The alteration in the plasma membrane results from the translocation of phosphatidylserine (PS) from the inner to the outer leaflet, where it is typically located on the cytoplasmic surface. Phosphatidylserine (PS) present on the cell surface exhibits a strong binding affinity for Annexin V. During the advanced phases of apoptosis and necrosis, cell membranes compromise their integrity, facilitating the entry of propidium iodide (PI) into the cell nucleus. Double staining with Annexin V and propidium iodide (PI) was employed to differentiate between cells in early apoptosis (Annexin V FITC+ and PI−), late apoptosis (Annexin V FITC+ and PI+), and necrosis (Annexin V FITC− and PI+). Cells that were negative for both markers (Annexin V FITC− and PI−) acted as controls.

**Figure 13:**
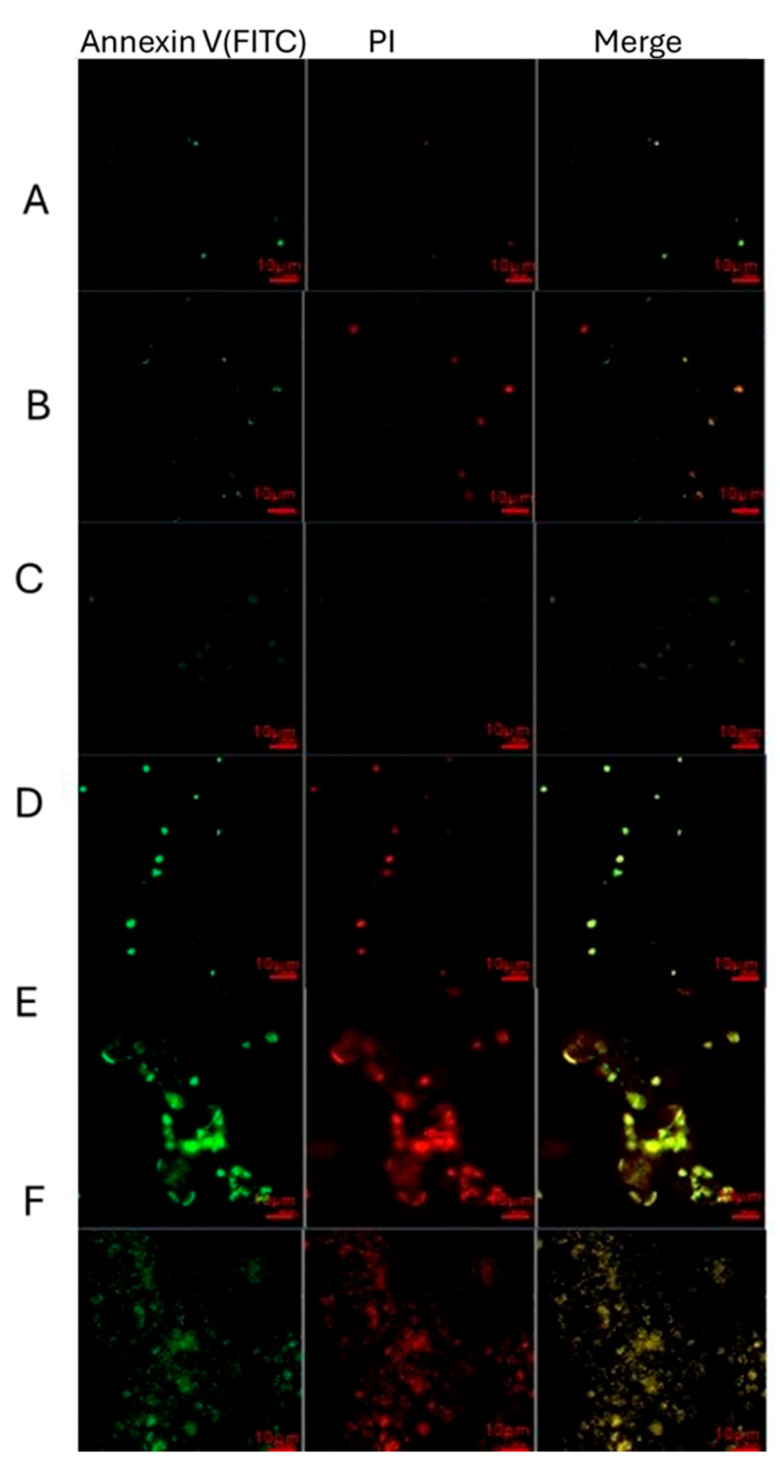
Apoptosis detection using fluorescence microscopy. Cells were treated with either control or Sn 10 nM + NPs and Lit in complete media for 24 h. Necrotic and apoptotic cells were identified through fluorescence microscopy (20×) after staining with Annexin V-FITC and PI. Annexin V-FITC was used to detect apoptotic cells, while PI was used to identify necrotic cells. ((**A**); Control, (**B**); Sn-38 10 nM, (**C**); Sn 10 nM + NPs 25 µg/mL + Lit 25 µg/mL, (**D**); Sn 10 nM + NPs 25 µg/mL + Lit 50 µg/mL, (**E**); Sn 10 nM + NPs 50 µg/mL + Lit 25 µg/mL, (**F**); Sn 10 nM + NPs 50 µg/mL + Lit 50 µg/mL).

Under fluorescence microscopy, cells stained with annexin V appeared green, while those stained with PI appeared red. After U373 cells were exposed to Lithium, Sn-38, and cerium oxide nanoparticles for 24 h, Figure 13 shows features of late apoptosis and necrosis. These findings confirm that changes in BAX/Bcl-2 levels occurred. Therefore, our study demonstrates that combined treatment with Lithium, Sn-38, and cerium oxide triggered apoptosis in U373 GBM cells (Figure 13).

### 2.7. Gene Results

**Figure 14:**
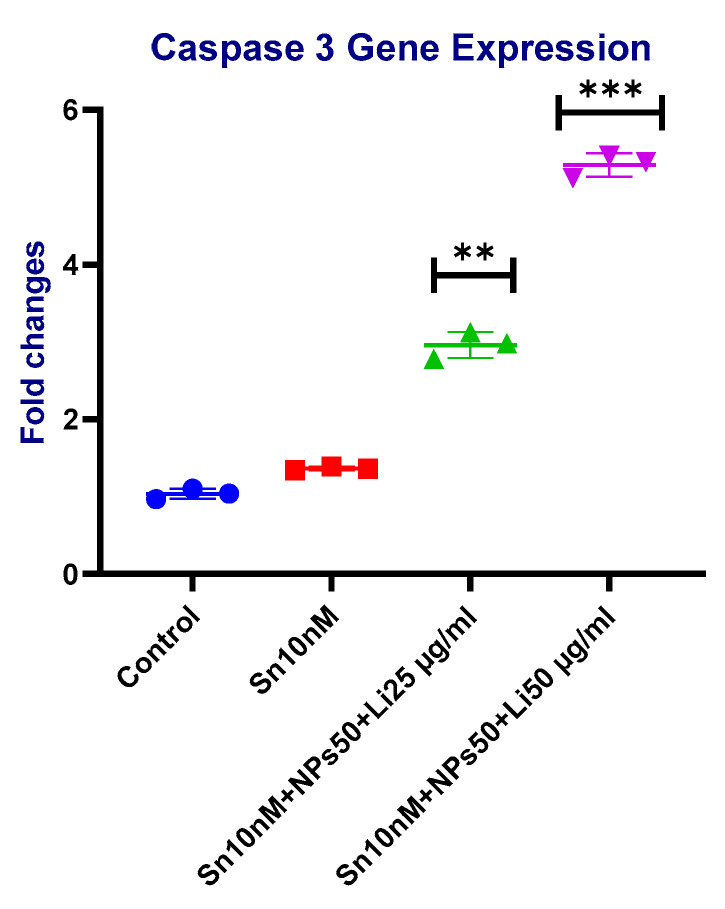
*Caspase-3* levels of control, Sn-38, NPs, and treatment groups. Sn 10 nM + NPs 50 µg/mL + Lit 25 µg/mL, and Sn 10 nM + NPs 50 µg/mL + Lit 50 µg/mL combined dose groups show a significant increase compared to the control (** *p* < 0.01, *** *p* < 0.001).

According to the *Caspase-3* gene expression results, there was approximately a 4-fold increase in the group treated with Sn-38 10 nM + NPs 50 μg/mL + Lit 25 μg/mL compared to the control. The most significant result, with about a 6-fold increase compared to the control, was observed in the group treated with Sn-38 10 nM + NPs 50 μg/mL + Lit 50 μg/mL (Figure 14).

**Figure 15:**
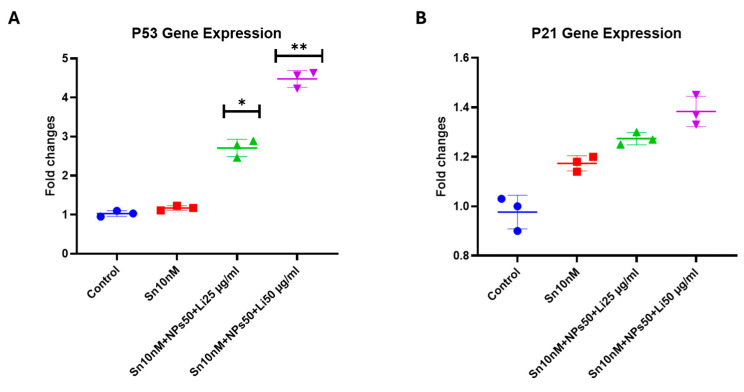
*p53* and *p21* gene expression levels of control, Sn-38, NPs, and treatment groups. (**A**); Sn 10 nM + NPs 50 µg/mL + Lit 25 µg/mL, and Sn 10 nM + NPs 50 µg/mL + Lit 50 µg/mL combined dose groups show a significant increase compared to the control. (**B**); No significant difference was observed in the *p21* gene expression level compared to the control (* *p* < 0.05, ** *p* < 0.01).

The *p53* gene expression levels are shown in Figure 15A. The results indicate that there was about a 1.5-fold increase in the group treated with Sn-38 10 nM + NPs 50 μg/mL + Lit 25 μg/mL compared to the control. The most significant result, with roughly a 3-fold increase over the control, was observed in the group treated with Sn-38 10 nM + NPs 50 μg/mL + Lit 50 μg/mL. The *p21* gene expression results are presented in Figure 15B. No notable difference in *p21* gene expression was observed compared to the control.

**Figure 16:**
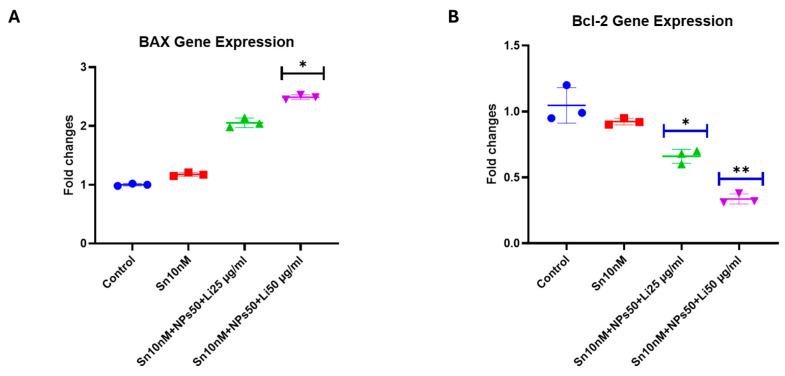
*BAX* and *Bcl-2* gene expression levels in control, Sn-38, NPs, and treatment groups. (**A**). Sn 10 nM + NPs 50 µg/mL + Lit 50 µg/mL combined dose group shows a significant increase compared to the control. (**B**). Sn 10 nM + NPs 50 µg/mL + Lit 25 µg/mL and Sn 10 nM + NPs 50 µg/mL + Lit 50 µg/mL treatment groups show a significant decrease compared to the control (* *p* < 0.05, ** *p* < 0.01).

The results for *BAX* and *Bcl-2* gene expressions are shown in Figure 16. According to our findings, *BAX* increased approximately 3-fold in the Sn-38 10 nM + NPs 50 μg/mL + Lit 50 μg/mL combined treatment group compared to the control. Conversely, Bcl-2 showed a significant decrease when compared to the Sn-38 10 nM + NPs 50 μg/mL + Lit 25 μg/mL and Sn-38 10 nM + NPs 50 μg/mL + Lit 50 μg/mL control.

### 2.8. Western Blot Results

**Figure 17:**
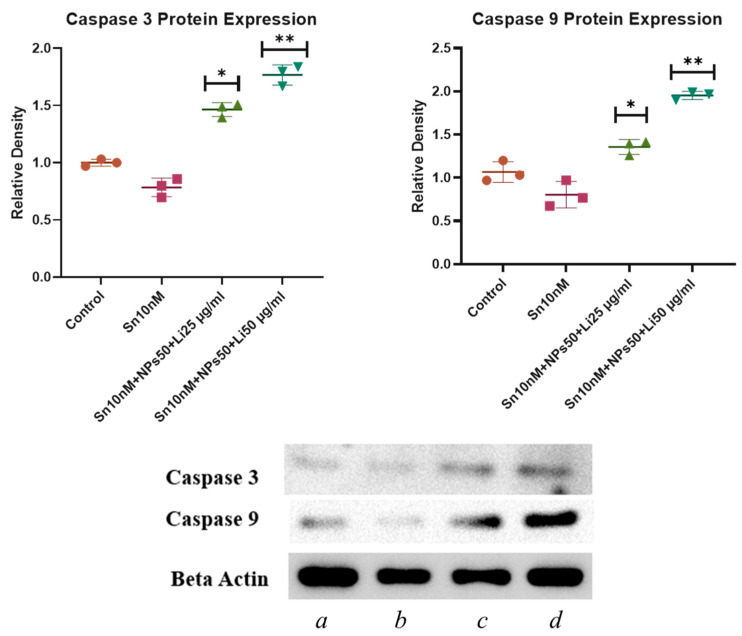
Increases in apoptotic marker expressions induced by lithium and Sn-38 treatment were modulated by Western blot analysis (Mean ± SD and *n* = 3). Protein bands of the expression levels of apoptotic markers Caspase-3 and Caspase-9, β-actin was used as a loading control protein (a; Control, b; Sn 10 nM, c; Sn 10 nM + NPs 50 µg/mL + Lit 25 µg/mL, d; Sn 10 nM + NPs 50 µg/mL + Lit 50 µg/mL) (* *p* < 0.05, ** *p* < 0.01).

Western blot results of Caspase-3, Caspase-9, and β-actin expressions are shown in Figure 17. Caspase-3 protein level, which is an apoptotic marker, showed a significant increase in the Sn 10 nM + NPs 50 µg/mL + Lit 25 µg/mL and Sn 10 nM + NPs 50 µg/mL + Lit 50 µg/mL combined treatment groups compared to the control. Caspase-9 protein level, correlated with Caspase-3, showed a significant increase in the Sn 10 nM + NPs 50 µg/mL + Lit 25 µg/mL and Sn 10 nM + NPs 50 µg/mL + Lit 50 µg/mL combined treatment groups compared to the control.

## 3. Discussion

SN-38, a chemical compound developed as an analog of irinotecan, is one of the agents used in chemotherapy. Recent studies have shown that SN-38 exhibits antitumor activity against various tumors [23]. In a study, SN-38 was found to have a higher cytotoxic effect on the U87-MG cell line compared to irinotecan [24]. However, the high cytotoxicity of SN-38 enhances its side effect profile. Therefore, it is necessary to improve its therapeutic efficacy and reduce side effects through targeted delivery using combination therapies or drug carrier systems. In one study, the combination of SN-38 and rabusertib in glioblastoma cells was found to synergistically disrupt metabolites associated with epigenetic adaptations, leading to cytotoxicity [25].

Lithium is an important drug approved for treating bipolar disorder, affecting processes including metabolism, neuronal communication, cell proliferation, and development. It has been suggested that lithium might effectively inhibit glioma invasion as a therapeutic strategy to prevent tumor recurrence or progression [26]. Consequently, a lower incidence of certain solid tumors has been observed in patients receiving lithium treatment [11].

Analysis of Ce_2_O_3_ nanoparticles indicates that particles suitable for biomedical applications were obtained. According to DLS results, the nanoparticles have an average Z-dimension of 213.4 nm and a PDI value of 0.38, with a main size population around 83.83 nm, indicating that the particles are generally in the nanometric range and exhibit a partially monodisperse distribution [27]. The fact that the main population is below 100 nm indicates that these nanoparticles have the potential to cross the blood–brain barrier (BBB). FTIR analysis confirmed the successful formation of the Ce_2_O_3_ structure with vibrational bands of O–H at 3400 cm^−1^, H–O–H at 1630 cm^−1^, CO_3_^2−^ at 1450–1400 cm^−1^, and Ce–O at 500–600 cm^−1^. These findings, consistent with the literature, indicate that the synthesized particles exhibit Ce–O lattice vibrations and surface hydroxyl and carbonate groups [28]. In conclusion, the synthesized Ce_2_O_3_ nanoparticles have the potential for neuroprotective and antioxidant applications due to their size, moderate monodispersity, and Ce–O characteristics suitable for BBB permeation [29,30].

Nanoparticles are often preferred as therapeutic agents, especially in brain cancers, because they can cross the blood–brain barrier and are biocompatible [31,32]. Numerous studies emphasize the neuroprotective, life-extending, and anti-inflammatory properties of cerium oxide. Developing new treatment strategies for GBM is essential. Identifying therapies that induce apoptosis is significant in this cancer type, which is known to resist apoptotic mechanisms. Thus, while our study showed significant effects of Ce_2_O_3_ nanoparticles on oxidative stress and apoptosis in glioblastoma cells, we did not provide direct experimental evidence that these nanoparticles cross the blood–brain barrier (BBB). However, the literature reports indicate that cerium oxide nanoparticles can cross the BBB, especially under oxidative or inflammatory conditions when endothelial integrity is compromised. Therefore, the effects seen in our in vitro glioblastoma model likely reflect neuronal uptake and redox modulation rather than physical barrier penetration. This neuronal uptake did not cause neurotoxicity in healthy neuronal cells. As a result, healthy neurons were spared, while GBM cells were vulnerable to oxidative damage and apoptosis.

Apoptosis is a cellular process triggered by irreparable damage that leads to controlled cell death. Factors like ROS accumulation or toxic substances cause DNA damage, prompting the cell to activate *p53* and repair mechanisms. Activated *p53* induces p21, arresting the cell cycle in the G1 phase to allow DNA repair [33]. DNA methylation, an epigenetic regulation mechanism, influences this repair process by regulating the levels of these proteins. If damage is unrepaired, *p53* activates *BAX* and inactivates *BCL-2* [34]. The imbalance between *BAX* and *BCL-2* facilitates pore formation in the mitochondrial membrane, leading to cytochrome C release. Cytochrome C then forms the apoptosome with *Caspase-9*, activating *Caspase-3*. Active Caspase-3 induces DNA damage and drives apoptosis. Based on the apoptotic mechanisms described in the literature, our study investigated the therapeutic effects of combining lithium, SN-38, and Ce_2_O_3_ nanoparticles on GBM, focusing on apoptosis-related inflammation and oxidative damage. Our results showed increased *BAX* levels in the SN-38 10 nM + NPs (50 µg/mL) + lithium (50 µg/mL) group compared to SN-38 alone and the control, with *BCL-2* levels decreasing in the same group, correlating with the increase in *BAX*. These findings support the study by Vredenburgh et al., which found that irinotecan treatment for GBM was associated with increased *BAX* and decreased *BCL-2* expression [35].

Additionally, we measured levels of *p53* and *p21* proteins, which are key indicators of apoptosis and methylation, through genetic analysis. *p21*, targeted by *p53*, arrests cells in the G1 phase. Similarly to *BAX*, *p53* and *p21* levels increased in the 10 nM + NPs (50 µg/mL) + Lit (50 µg/mL) group compared to the SN-38 group and control. This suggests suppression of DNA methylation, with elevated *p53* and *p21* levels driving cells toward apoptosis. Literature, including Lu et al., reports that combined SN-38 and imatinib treatment in glioma increases *p53* and *p21* levels [36]. The levels of *p53* and *p21* observed in our results support this. Apoptosis was also assessed using the Annexin V-FITC and PI staining method. These groups were stained, and early apoptosis (Annexin V-FITC) and late apoptosis (PI) were detected via IF imaging. Apoptosis increased with higher doses, especially in the SN-38 10 nM + NPs 50 µg/mL + Lit 50 µg/mL group, which exhibited more intense staining fluorescence, indicating greater cell death. This supports the role of SN-38 and NPs in GBM treatment reported in the literature [37].

The regulation of apoptosis involves multiple cellular processes. For instance, *p53*-activated PTEN inhibits the PI3K/AKT/mTOR pathway, preventing cell proliferation. Therefore, PTEN activation is crucial in GBM and other cancers [38]. In our study, PTEN levels increased in treatment groups with dose escalation, aligning with studies showing PTEN levels rise with therapeutic effects [39]. The PI3K/Akt/mTOR pathway influences cancer development by regulating key processes such as apoptosis, transcription, translation, metabolism, angiogenesis, and cell cycle progression. In GBM, this pathway is often regulated [40,41]. Our data show decreased levels of PI3K, AKT, mTOR, STAT-3, and MMP-2 in treatment groups as doses increased, approaching control levels. Literature indicates that Apitolisib, a PI3K/mTOR inhibitor tested on GBM, induces apoptosis [42]. Conversely, polymeric nanoparticles loaded with SN-38 have been reported to support apoptosis in colorectal cancer by modulating the same pathway [19].

Excess ROS accumulation causes cellular damage, prompting apoptosis [43]. A study using U251 cells treated with resveratrol found increased ROS levels and decreased SOD-2, an antioxidant enzyme, which leads to apoptosis [44]. Oxidative damage also activates inflammatory molecules, mainly regulated by interleukins. Oxidative stress can promote cell proliferation and inhibit apoptosis by inducing the secretion of IL-1β. When Temozolomide (TMZ) was administered to U87-S cells, cell migration decreased, while levels of IL-1β and IL-6 increased significantly [45]. Consistent with these findings, our study found that combining SN-38, lithium, and CeO_2_ nanoparticles led to increased oxidative damage markers, such as IL-1β, indicating inflammation. These results were supported by apoptosis assays, which showed elevated levels of *p53* and p21, suggesting that the cells were unable to repair the damage and subsequently underwent apoptosis. Furthermore, we observed a decrease in PI3K, AKT, mTOR, STAT-3, and MMP-2, which are associated with cell survival and migration, while PTEN levels increased, highlighting the therapeutic potential of this combination. In conclusion, our study demonstrates that the combination of SN-38 with lithium and CeO_2_ nanoparticles, particularly at the dose of SN-38 10 nM + Li 50 µg/mL + NPs 50 µg/mL, exerts promising therapeutic effects on the GBM cell line U373.

## 4. Materials and Methods

### 4.1. Hydrothermal Synthesis and Characterization of Cerium Oxide NPs

Ce_2_O_3_ nanoparticles were synthesized by the hydrothermal synthesis method in Cerium (III) nitrate (Ce (NO_3_)_3_) Na-citrate medium. For this purpose, the Polat and Nadaroglu method was modified [46]. Briefly, Cerium (III) nitrate hexahydrate [Ce (NO_3_) _3_·6H_2_O] (Sigma-Aldrich, Burlington, MA, USA, ≥99% purity) was used as the cerium source, and sodium citrate (Na_3_C_6_H_5_O_7_) served as both a stabilizing and capping agent, preventing agglomeration during nucleation and growth. A 0.1 M aqueous solution of Ce (NO_3_)_3_ was prepared, and Na-citrate was added under continuous magnetic stirring at room temperature (25 °C) for 30 min until a clear, homogeneously mixed solution was obtained. After adding Na-citrate to the Cerium (III) nitrate solution and mixing it well at room temperature, it was transferred into the steel reactor and reacted at 200 °C for 8 h. To ensure complete removal of ethanol, the washed precipitate was dried in a ventilated oven at 60 °C for 12 h. Additionally, before conducting biological experiments, the dried nanoparticle powder was re-suspended in sterile deionized water and left open to air at room temperature for 24 h to allow for the total evaporation of any remaining ethanol traces [27,47]. Morphological characterization of Ce_2_O_3_ NPs was performed by Scanning Electron Microscope (SEM). For this purpose, Bilecik Şeyh Edebali University established the Central Research Laboratory Application and Research Center (BARUM). For this purpose, the ZEISS/Supra 40 VP Scanning Electron Microscope (SEM) was used (ZEISS, Oberkochen, Germany). Zeta potential measurements were analyzed using the MALVERN/NANO-ZS Zeta potential device (Malvern, Worcestershire, UK), and FT-IR measurements were analyzed using the PERKIN ELMER/SPECTRUM 100 (Perkin Elmer, Shelton, WA, USA).

### 4.2. Obtaining the Bioconjugate Drug and Determining the Drug Release Profile

The bioconjugate drug formulation was prepared using the electroporation technique. For this purpose, 5 mg of nanoparticles (NPs) were accurately weighed and combined with 10 nM of SN-38 (5 mg) and lithium (5 mg). The mixture was dissolved in 1 mL of phosphate-buffered saline (PBS) and transferred into an electroporation cuvette. Subsequently, the sample was subjected to short-duration electric pulses (10 pulses) at optimized conditions of 99 V and 10 ms per pulse. This process facilitated the encapsulation of the drug within the nanoparticle carrier system, resulting in the formation of a bioconjugate structure. The obtained bioconjugate dispersion was then diluted in 100 mL of distilled water and incubated on a shaker for 72 h to ensure complete stabilization.

The drug release profile was evaluated using a UV–Vis spectrophotometer (Thermo Fisher Scientific, Waltham, MA, USA) by measuring absorbance at predetermined time intervals ranging from 0 to 72 h. The release kinetics were analyzed based on the cumulative drug release over time to determine the release mechanism and rate constants [48,49].

### 4.3. Cell Culture

U373 GBM and SHSY-5Y cell line was obtained from Bilecik Seyh Edebali University, Department of Medical Pharmacology (Bilecik, Turkey). Growth and development of cells were carried out with Dulbecco’s Modified Eagle Medium supplemented with 10% Fetal Bovine Serum (Euroclone, Milan, Italy) and F-12 medium (Euroclone, Milan, Italy) containing 0.1% Penicillin/Streptomycin. Cells were grown at 37 °C in an environment containing 5% CO_2_ [50].

### 4.4. Wound Assay

The migration rate of treatment groups was evaluated in the U373 GBM cell line by the wound test. U373 cells were seeded in a 96-well plate and incubated until 100% confluence. Then, each well was injected with a sterile plastic pipette tip (yellow tip-200 µL) to create a wound model. Cell debris was aspirated to be with PBS. The cells were then exposed to various treatment concentrations. Images taken from the central region of the wound were photographed every 0–48 h to evaluate cell migration. All experiments were repeated three times [50].

### 4.5. Cellular Therapy

After the cells reached sufficient density, they were passaged and seeded in 96-well plates. Group cells were treated with specific concentrations of SN-38 10 nM [5,6,7], Lithium, and SN-38+ Lithium combinations, and SN-38+ Lithium+ NPs. The drug amounts used in all combined treatment groups represent the final concentrations. The concentrations of SN-38 (10 nM), lithium (50 µg/mL), and CeO_2_ NPs (50 µg/mL) were selected based on prior literature and preliminary dose–response experiments to achieve pharmacologically relevant yet non-toxic conditions. These doses have been previously reported to modulate apoptosis-related signaling in glioma cells and were therefore used to evaluate synergistic effects in this study. The groups are listed in detail in Table 1 below.

### 4.6. Cell Viability (MTT) Test

The MTT assay was conducted using a commercially available kit from Sigma-Aldrich, USA. In summary, MTT (10 µL at 5 mg/mL) was added to each well and incubated for 4 h at 37 °C in a 5% CO_2_ atmosphere. Subsequently, dimethyl sulfoxide (DMSO) was added to each well to dissolve the formazan crystals. Cell viability (%) was determined by measuring the optical density (OD) at 570 nm with a spectrophotometer. The spectral density of the control group was established at 100 (*n* = 8) [51].

### 4.7. Analysis of Oxidative Stress Biomarkers (TOS and TAC)

The TOS assay has been evaluated analytically, depending on the number of oxidants in the sample. The initial absorbance of the sample-buffer mixture was measured at 530 nm. Subsequently, the prochromogen solution was introduced, and the second absorbance measurement was recorded at 530 nm. The antioxidant capacity was assessed by preventing the generation of the 2,2’-azinobis(3-ethylbenzothiazoline-6-sulfonate) (ABTS+) radical cation. TAC was measured qualitatively utilizing the Total Antioxidant Status Kit. The original wavelength of the sample-buffer mixture was recorded at 660 nm. Following that, the ABTS radical solution was introduced, and after 10 min, the second measurement was conducted at 660 nm [51] (repeated so that *n* = 8).

### 4.8. ELISA Tests

To test the effects of the substances used on inflammation, apoptosis, and anti-apoptosis mechanisms, IL-1β (SunRed, LOT; 202305), BAX (BT-LAB, LOT; 202311011), BCL-2 (BT-LAB, LOT; 202311011), PTEN (BT-LAB, LOT; 202408010), AKT (BT-LAB, LOT; 202408016), mTOR (BT-LAB, LOT; 202408010), STAT-3 (BT-LAB, LOT; 202408010), and MMP-2 (BT-LAB, LOT; 202108007) levels were evaluated using the ELISA kit. The analysis was repeated three times. The experiment was performed according to the kit protocol (during the experiment, cells were incubated for approximately 1 h according to the protocol), and each sample’s optical densities were measured at 450 nm [51]. The analysis was repeated three times.

### 4.9. Annexin V-FITC Immunofluorescent Stain

The ApoDETECT Annexin V-FITC Kit (Thermo) was used to measure the annexin V-PI level. Each sample had between 105 and 106 cells aliquoted, and the cells were centrifuged for five minutes at 400× *g*. After being decanted, the supernatant was rinsed with chilled PBS (pH: 7). After being carefully suspended, the pellet was centrifuged again. They decanted the supernatant. An equivalent volume of deionized water was used to dilute the binding buffer until the final volume had a 1X concentration (10 mM Hepes/NaOH, pH 7.4, 140 mM NaCl, 2.5 mM CaCl_2_). 190 microliters of 1X binding buffer were used to resuspend the cells. After adding 10 µL of Annexin V-FITC to the cell solution, it was allowed to sit at room temperature for 10 min. After giving the cells another wash with a binding buffer, they were centrifuged for five minutes at 400× *g*. 190 µL of 1X binding buffer was used to resuspend the supernatant after it had been decanted. After adding 10 µL of a stock solution containing 20 µg/mL propidium iodide, pictures were captured using an immunofluorescence microscope set at 20× magnification [52]. The analysis was repeated three times.

### 4.10. Gene Analysis

Total RNA isolation from U373 GBM cells was performed by using the EcoPURE RNA isolation kit (EcoTECH Biotecnology, Erzurum, Turkey) according to the manufacturer’s product manual. cDNA synthesis was performed with a cDNA synthesis kit with a fixed amount of RNA from each sample (EcoScript ). *Caspase-3*, *p21*, *p53*, *BAX*, and *Bcl-2* gene expression (Table 2) levels were analyzed with Real-Time PCR, LightCycler^®^ 96 using ClearPeak 2x SYBR Master Mix. Cells were studied in 3 replicates. The annealing temperature of all gene regions was 60 °C. The gene expression levels were estimated as (2^−ΔΔCt^) [53].

### 4.11. Western Blot Analysis

Spectrophotometric measurements were made using the BCA Protein Assay Kit (Thermo Fisher Scientific, Cat# 23227) to determine the total protein content in the supernatants obtained from U373 glioblastoma (GBM) cells. Equal quantities of protein (50 µg per well) were applied to SDS-PAGE gels and separated utilizing the Bio-Rad Mini-PROTEAN^®^ Tetra Cell electrophoresis apparatus. Following electrophoresis, the proteins were transferred to nitrocellulose membranes (NC) via a semi-dry transfer apparatus (Bio-Rad, Hercules, CA, USA). A sandwich assembly was created for the transfer in the following sequence: sponge, filter paper, gel, nitrocellulose membrane, filter paper, and sponge (from cathode to anode). Following transfer, the membranes were blocked and subsequently incubated overnight at 4 °C with primary antibodies diluted in 5% (*w*/*v*) nonfat milk in TBS-T (Tris-buffered saline with 0.1% Tween 20). The main antibodies used were β-actin (1:1000), Caspase-3 (1:1000), and Caspase-9 (1:1000). Secondary antibodies appropriate for the primer were diluted in 5% milk powder (Anti-Mouse Secondary Antibody: 1/5000, Anti-Rabbit Secondary Antibody: 1/10,000), and the membranes were treated with these antibodies for 1 h. Membranes were subjected to treatment with a chemiluminescent conjugate (ECL). Band pictures were acquired via the SYNGENE G: Box Chemi XRQ imaging apparatus. Band intensities in the acquired images were quantified using ImageJ 2. Protein expression levels were standardized to β-actin, an internal control, and represented as a percentage of the control [54]. The analysis was repeated three times.

### 4.12. Statistical Analysis

Statistical evaluations within groups were conducted with one-way ANOVA. All calculations were performed using SPSS 20 for statistical analysis, with *p* < 0.05 considered statistically significant for all tests. Results are expressed as the mean and standard deviation (mean ± SD).

## 5. Conclusions

In this study, we investigated the combined effects of lithium, SN-38, and cerium oxide nanoparticles (Ce_2_O_3_ NPs) on glioblastoma cells to assess their effects on cell viability, apoptosis, and ROS production. Our findings indicate that both SN-38+Li and SN-38+Li+Ce_2_O_3_ NP combinations significantly enhanced anticancer activity through the induction of apoptosis, suppression of proliferation, and modulation of oxidative stress. The increased therapeutic efficacy was associated with changes in the MMP-2, PTEN, AKT, mTOR, STAT-3, and Bax/Bcl-2 signaling pathways. Importantly, Ce_2_O_3_ NPs alone exhibited superior biocompatibility, supporting their potential as safe nanocarriers for drug delivery.

Future Recommendations: Further in vivo studies and mechanistic analyses are recommended to confirm these findings, optimize dosing strategies, and investigate the long-term effects on oxidative signaling, mitochondrial function, and neuroinflammation, thereby strengthening the translational potential of the SN-38–Li–Ce_2_O_3_ NP combination for the treatment of glioblastoma.

### Study of Limitations

This study has certain limitations. The findings are based solely on in vitro experiments using the U373 glioblastoma cell line and may therefore not fully reflect in vivo conditions. In addition, validation across different cell lines and animal models was not performed, nor were comprehensive dose–response and long-term evaluations conducted. The study primarily focused on specific mechanisms, while the nanoparticles’ long-term stability was not thoroughly investigated. Therefore, to increase the reliability and generalizability of the findings, they must be supported by in vivo studies.

## Figures and Tables

**Table 1:** List of treatment groups and doses.

SN-38 and Lithium Groups	SN-38, Lithium, and NPs Groups
Control	Control
Sn-38 10 nM	Sn-38 10 nM
Lit 1 µg/mL	NPs 25 µg/mL
Lit 5 µg/mL	NPs 50 µg/ml
Lit 10 µg/mL	Sn-38+NPs (25 µg/mL)+Li 25 µg/mL
Lit 25 µg/mL	Sn-38+NPs (25 µg/mL)+Li 50 µg/mL
Lit 50 µg/mL	Sn-38+NPs (50 µg/mL)+Li 25 µg/mL
Sn-38+Lit 5 µg/mL	Sn-38+NPs (50 µg/mL)+Li 50 µg/mL
Sn-38+Lit 10 µg/mL	-
Sn-38+Lit 25 µg/mL	-
Sn-38+Lit 50 µg/mL	-

**Table 2:** List of gene PRIMER table.

Genes	Forward	Reverse
*Caspase-3*	5′ AGCGAATCAATGGACTCTGGA 3′	5′ TTCCCTGAGGTTTGCTGCAT 3′
*P-21*	5′ GACCTGTCACTGTCTTTGTAC 3′	5′ CTCTCATTCAACCGCCTAG 3′
*P-53*	5′ CCACCATGAGCGCTGCTCA 3′	5′ GCAGGGGAGGGAGAGATG 3′
*Bax*	5′ TTCATCCAGGATCGAGCAGG 3′	5′ GGAAAAAGACCTCTCGGGGG 3′
*Bcl-2*	5′ CCTGTGGATGACTGAGTACC 3′	5′ GAGACAGCCAGGAGAAATCA 3′

## Data Availability

The original contributions presented in this study are included in the article. Further inquiries can be directed to the corresponding author. (The data are not publicly available due to privacy or ethical restrictions).

## Abbreviations

The following abbreviations are used in this manuscript:

AKT	Serine/threonine kinase
GBM	Glioblastoma
GSK3β	Glycogen Synthase Kinase 3β
Lit, Li	Lithium
MMP-2	Matrix metalloproteinase-2
mTOR	Mammalian Target of Rapamycin
NPs	Nanoparticle
PKC	Protein kinase C
PTEN	Protein tyrosine phosphatase and tensin homolog
STAT-3	Signal transducer and transcription activator 3
TAC	Total Antioxidant Capacity
TOS	Total Oxidative Stress
